# A Preliminary Sequence Analysis of the Epstein-Barr Virus Nuclear Antigen 1 (EBNA1) Carboxy-Terminal Region in Cervical and Ovarian Cancers

**DOI:** 10.30699/ijp.2023.551761.2872

**Published:** 2023-03-23

**Authors:** Fatemeh Hosseini Tabatabaie, Seyed Younes Hosseini, Seyed Mohammad Ali Hashemi, Akbar Safaie, Jamal Sarvari

**Affiliations:** 1 *Department of Bacteriology & Virology, School of Medicine, Shiraz University of Medical Sciences, Shiraz, Iran *; 2 *Department of Pathology, School of Medicine, Shiraz University of Medical Sciences, Shiraz, Iran *; 3 *Gastroenterohepathology Research Center, Shiraz University of Medical Sciences, Shiraz, Iran*

**Keywords:** Cervical cancer, EBNA1, Epstein-Barr virus, Mutation, Ovarian cancer

## Abstract

**Background & Objective:**

Epstein-Barr virus nuclear antigen-1 (EBNA1) is one of the most important proteins of Epstein-Barr virus (EBV) that might be mutated in various related cancers. The purpose of this study was to compare EBNA1 mutations in the C-terminal region between patients with cervical and ovarian cancer and healthy individuals.

**Methods:**

As test and control groups, 18 EBV-positive paraffin-embedded samples of cervical and ovarian cancer and 10 age- and gender-matched healthy volunteers who did not have cancer but were EBV-positive were both used. Utilizing a commercial DNA extraction kit, total DNA was extracted following deparaffinization. The entire C-terminal region of the EBNA1 sequence was amplified using an in-house nested PCR. Phylogenetic analysis and Sanger sequencing were used to analyze the sequences using MEGA 7 software and through NJ method.

**Results:**

Sequence analysis revealed that the P-Ala subtype of EBNA1 was present in all samples. In two and one samples, respectively, of cervical cancer patients, the mutations A1887G and G1891A were found. The G1595T mutation was also detected in four sequences taken from ovarian cancer patients. No statistically significant difference could be found between the frequency of mutations in patients and controls (*P*>0.05). No known amino acid substitutions were found in the USP7-binding region and the DBD/DD domain.

**Conclusion:**

The findings showed that P-Ala is the predominant EBV subtype across all samples. Additionally, as the sequence of EBNA1's C-terminal region is so stable, it's possible that it had little impact on the pathogenesis of ovarian and cervical malignancies. It is advised to conduct additional research to verify these findings.

## Introduction

The incidence and death of cancer are increasing dramatically on a global scale. In many countries, cancer ranges from the first to the fourth most common cause of mortality before the age of 70 (1). Cervical adenocarcinoma is the fourth most commonly diagnosed cancer in women worldwide and the leading cause of cancer death in 36 countries (1, 2). According to studies, 600,000 and 300,000 people are diagnosed with and die from cervical cancer each year, respectively (3). In 2012, 370 deaths were reported due to cervical cancer in Iran(4). In a review study, the mortality rate from this cancer was reported to be 42% (5). Ovarian cancer has also one of the worst prognoses with a high mortality rate around the world (6). Globally, there are 295,414 new cases and 184,799 deaths related to ovarian cancer each year (7).

Viral infections, such as papillomaviruses (HPVs), Epstein-Barr virus (EBV), hepatitis B virus (HBV), hepatitis C virus (HCV), human T-lymphotropic virus type 1 (HTLV-1), and Merkel cell polyomavirus (MCPyV), are responsible for about 15–20% of cancer cases (8). EBV, also known as human herpes virus-4 (HHV4), which is a subset of gamma-herpesviruses, accounts for 38% of virus-associated cancers (9). This virus is frequently transmitted by oral secretions, blood transfusions, and organ transplants (10). It is responsible for infectious mononucleosis in 35-70% of adolescents and young adults (11). 

Although the role of EBV in some cancers such as Hodgkin's lymphoma, nasopharyngeal carcinoma, Burkitt's lymphoma, and gastric cancer is cleared, the exact pathogenesis of EBV in cervical and ovarian cancer remains unknown (12). Today, the main cause of cervical cancer is the human papillomavirus (HPV), mostly HPV-16 and -18 (~70%) (13). However, some studies have shown a possible link between EBV and cervical and ovarian cancer (14, 15).

Although CD21 and HLA-II serve as the major receptor and co-receptor for EBV entrance into B-cells, respectively, epithelial cells do not express CD21 receptors. The direct infection of EBV in the epithelial cells may occur through an unidentified receptor or by close cell-to-cell contact (16). Moreover, there is a hypothesis that epithelial cells might become infected through direct contact with infected B-cells (17).

Suppression of the viral lytic phase and increased expression of latent proteins lead to immortality and transformation of the epithelial cells (18). It is possible that EBV could transform cervical cells via the CD21 receptor, making these cells more susceptible to oncogenic antigens (9). It has also been shown that factors such as increasing age and progression of cervical lesions to a higher degree can increase the risk of EBV infection (9). Although it is uncertain whether EBV alone or in combination with HPV causes cervical cancer, there is evidence that EBV may speed up the integration of HPV's genome. According to Khenchouche* et al.*, squamous cell carcinoma (SCC) and cervical intraepithelial neoplasia (CIN-II and III) samples have been shown to have considerably higher HPV/EBV co-infection rates than CIN-I and normal cases (19). 

EBNA1 is expressed in the lytic phase and all EBV latency types except latency type 0. EBNA1 is the only viral protein involved in DNA replication in latency and is expressed in all EBV-related tumors (20, 21). Some reports showed that the expression of EBNA1 increased the tumorigenicity of EBV-negative tumors (20). Also, EBNA1, through interaction with Ori-P, helps the virus maintain the episomal form of the genome in cells and increase its transcription (22). EBNA1 can decrease apoptosis and DNA repair in EBV-associated lesions and probably has an important role in the transformation of EBV and HPV-infected lesions (23, 24).

The important domains of the EBNA1 protein include two Gly-Arg-rich regions, a Gly-Ala repeat, a ubiquitin-specific peptidase 7 (USP7) binding site, and DNA binding and dimerization domain (DBD/DD) (20). The DBD/DD domain is crucial for viral genome replication because deletions in specific regions of EBNA1, particularly the amino acids 395 to 450, promote virus replication (20). USP7 is a cellular protein that can bind to Human Herpes Virus Infected Cell Polypeptide 0 (ICP0) of Herpes simplex virus-1 (HSV-1). It is shown that USP7 has a tumor-suppressive effect by increasing the half-life of p53 and oncogenic properties that are affected by mouse double minute 2 homolog (MDM2) activity (25). Some theories state that the USP7-binding site of EBNA1 could affect p53 degradation by blocking its interaction with USP7 (20). According to previous studies, the detection rate of EBNA1 was higher in cervical carcinoma than in non-carcinoma tissues (24). 

So far, based on the 487 amino acids of EBNA1, five EBV subtypes with different geographical frequencies have been reported (26). Some studies showed that EBNA1 subtypes, especially V-Val, could change transcriptional activation properties in epithelial cell lines (27-29). The V-Val subtype's DBD/DD domain is likely linked to increased tumor cell proliferation (30). The diversity of the dominant EBNA1 subspecies in each country indicates its geographical dependence.

Therefore, this study aimed to compare the C-terminal region of EBNA1 mutations in cervical and ovarian cancer patients to those in the healthy control group.

## Material and Methods

**Study Population**

In this case-control study, 18 EBV-positive paraffin-embedded biopsy samples, including nine cervical and nine ovarian cancers, which were considered positive for EBV in the previous study, were enrolled (31). The samples were selected based on the pathology report from the biopsy bank of Shahid Faghihi Hospital and Shahid Motahari clinic, affiliated with Shiraz University of Medical Sciences, from 2014 to 2017. A specialized pathologist once more examined the samples. Moreover, 10 age- and gender-matched healthy, non-cancerous/EBV-positive volunteer subjects were enrolled as the control group. Whole blood was taken randomly from the control group, and sampling was continued until 10 EBV-positive samples were obtained. The Shiraz University of Medical Sciences Ethics Committee approved the study (IR.SUMS.REC.1399.948), and healthy volunteer subjects' informed agreement was obtained prior to sample collection.

**Deparaffinization, DNA Extraction, and DNA Integrity Assay**

Ten thick sections (10 μm) of paraffin-embedded tissue blocks from each patient were cut and collected in separate 2 mL autoclaved Eppendorf micro-centrifuge tubes and subjected to the deparaffinization step, as described previously (32). Following the manufacturer's instructions, DNA was extracted by a QIAamp DNA mini kit (Qiagen, Düsseldorf, Germany). Moreover, 10 mL of whole blood was collected from each healthy control subject and put into 750 µL EDTA tubes. After centrifugation, the buffy coat was removed and dispersed in 1000 µL of red cell lysis buffer (Cytomatingene, Isfahan, Iran). The second centrifugation was done at 500 ×g for 10 min at room temperature. After discarding the liquid, the leukocyte pellet was twice dissolved in 2 mL of PBS. The tubes were centrifuged, and the supernatants were discarded. DNA was extracted using the AnaCell kit (AnaCell, Tehran, Iran), according to the manufacturer's instructions. All extracted DNA was stored at -20°C until further processing. The quantity of extracted DNA was determined by Nanodrop (Nanodrop^TM ^Spectrophotometer, Thermo Scientific, USA). Moreover, DNA quality was assayed by a beta-globin gene PCR assay using PCO3 and PCO4 primers (Table 1), as described previously (33). 

**Nested-polymerase chain reaction assay:**

In order to amplify the EBNA1 gene fragment of each sample, we designed two pairs of primers using AlleleID software (Table 1). 

**Table 1:** The sequences, target location, and product length of each primer pair used in the study

Length of the product (bp)	Location	Sequences (5' 3')	Primers	Target
**110**	62170-62190	ACACAACTGTGTTCACTAGC	PCO3	β-Globin
	62299-62279	CAACTTCATCCACGTTCACC	PCO4	
**298**	1000-1019	TACTCCTTACTATGTTGTG	EBV-F	EBV detection (BHRF1 gene)
	1315-1297	CCTTGCCTAATATCCTAC	EBV-R	
**743**	1152-1173	CAGTAGTCAGTCATCATCATCC	Inner Forward	EBV sequencing (partial EBNA1)
	1894-1875	CACCTCCTTCATCTCCGT	Inner Reverse	
**806**	1092-1109	GAAGTCGTGAAAGAGCCA	Outer Forward	
	1896-1879	ATCACCTCCTTCATCTCC	Outer Reverse	

Each step of nested PCR was performed with a total of 1 µL DNA template, 10 µL MasterMix (Amplicon, Denmark), 0.5 µM each primer, and 8 µL DNase-free water (Sinaclon, Iran). Thermal cycling was initiated at 95°C for 7 min, followed by 30 cycles of denaturation at 95°C for 30 sec, annealing at 60°C for 35 sec, extension at 72°C for 45 sec, and a final extension at 72°C for 10 min. 1 µL of 1/100 dilution of the first round of products was used for the second round of amplification. The annealing step was performed using the touch-down technique in a temperature gradient from 63°C to 59°C. Finally, the PCR products were separated on 1.5% agarose gel and visualized by safe stain under UV light. To ensure the reliability of the PCR assay, in each run, we also included the extract from the B95 EBV positive cell line, as described before (34).

**DNA Sequencing**

PCR products were purified using a Top DNA purification kit (Topaz Gene, Tehran, Iran) according to the manufacturer's instructions. Then, the chain termination-based Sanger sequencing method was used to sequence the PCR products of the EBNA1 gene. Purified PCR products with an inner forward primer were subjected to single-read sequencing using the Genetic Analyzer Upgrade Kit (Thermo Fisher Scientific, USA) in the ABI 3500XL (Applied Biosystem, Foster City, USA) Genetic Analyzer. The sequencing results of EBNA1 were then checked by Chromas 2.6.4 (Technelysium) to find out if there were any nucleotide substitutions. The final results were retrieved by Molecular Evolutionary Genetics Analysis (MEGA) software, version 7.0. Then, the results were compared with several reference sequences. Reference sequences were obtained from the NCBI homepage (https://www.ncbi.nlm.nih.gov/), which included NC_009334, NC_006146, and NC_007605.

**Phylogenetic Analysis and Genotyping**

**Fig. 1:**
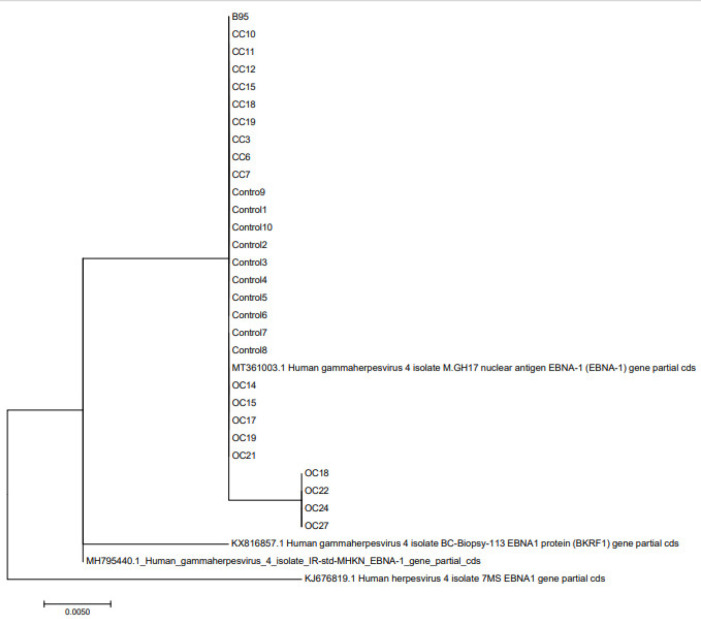
Phylogenetic tree analysis of sequences; the comparison of sequences from patients and healthy individuals indicated their close relationship with B95, as shown in a cluster

Following sequence analysis, the obtained sequences were multiple-aligned in MEGA7 software using the ClustalW method's default parameters with EBV EBNA1 reference sequences. Then, using MEGA7 software, the phylogenic tree was created using the neighbor-joining method concerning the Kimura-2 parameter-based distance estimate model (Figure 1). Finally, the designed tree was evaluated by 500-replicate bootstrapping to ensure its fidelity. All isolated sequences were checked for accuracy using references to form a homologous database, which was then used as the input for MEGA software's multiple alignments and NJ algorithm-based tree reconstruction (bootstrap replicate: 1000).

**Statistical Analysis**

Statistical analysis was performed through Epi Info version 7.1.5 (Center for Disease Control, Atlanta) using the Chi-square test. A P-value of < 0.05 was considered significant**.**

## Results

The patients' and healthy control groups' mean age was 56.1±3.9 and 54.7±4.5, respectively, which was not statistically significant (*P*=0.837). Furthermore, the mean age of cervical and ovarian cancer patients was 63.5±5 and 46.2±5, respectively, which was statistically insignificant (*P*=0.051). Also, statistical analysis showed that the mean age of cervical cancer patients, ovarian cancer patients, and healthy volunteer groups was not significantly different (*P*=0.111). In the cervical cancer group, all of the specimens were of the squamous cell type; two samples (22.2%) had CIN I, three (33.3%) had CIN II, one (11.1%) had CIN III, and also three of them (33.3%) had invasive SCC. Of the ovarian cancer patients, two cases (22.2%) were grade I, five (55.5%) were grade III, and two (22.2%) were identified to be in the early stages. Regarding the histopathological characteristics, eight (88.9%) ovarian cancer patients were diagnosed with serous adenocarcinoma and one (11.1%) patient with mucinous adenocarcinoma. Among nine samples from cervical cancer patients, seven were HPV positive.

**Fig. 2:**
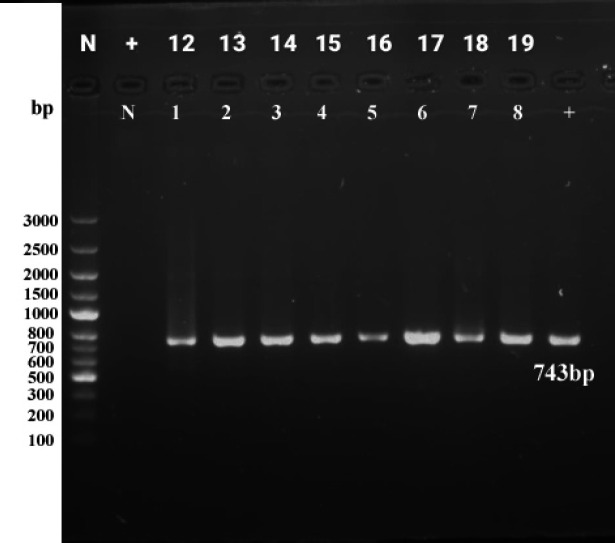
PCR gel electrophoresis analysis of samples from patients and controls. A pure 743 bp band have detected in all positive samples. N: Negative control, 1-3: Cervical cancer biopsies, 4-6: Ovarian cancer biopsies, 7-8: Healthy controls, +: Positive control

**Fig. 3:**
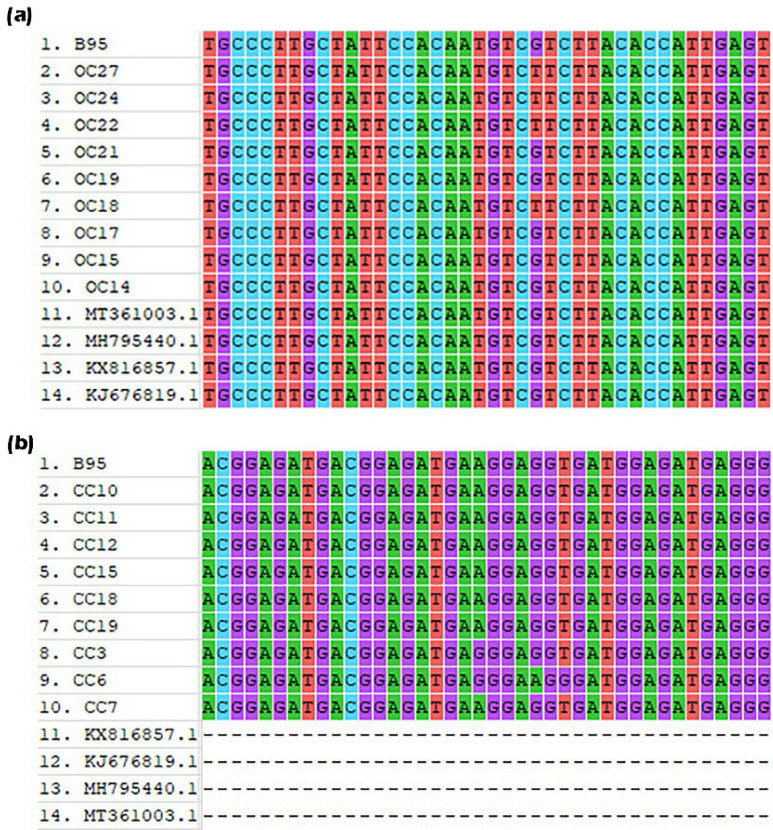
The multiple sequence alignment of ovarian cancer (a) and cervical cancer (b) samples with the reference sequences showed homogeny and a stable trend of the EBNA1 gene in this region. B95, MH795440.1, KJ676822, KX816857, and MT361003 have been used as reference sequences

All the PCR products in nested PCR had the same size and pattern on agarose gel, as shown in Figure 2. Sanger sequencing revealed that all patients and healthy control samples contained alanine residue at the 487 sites; therefore, the EBNA1 subtype was identified as P-Ala in all individuals, and no differences were observed between EBV isolates (Figure 3). Each isolate was compared with the reference and isolated Iranian sequences at the carboxyl terminus of the previously reported EBNA1 gene (MH795440.1, KJ676822, KX816857, and MT361003). Phylogenetic analysis showed an identical pattern between the sequences and the reference sequence B-95, indicating that all samples contained the EBV B95 strain.

There was no difference in the mutation frequency between patients and healthy samples (*P*>0.05). Sequence analysis of DBD/DD domains binding to cellular USP7 in the C-terminal region of EBNA1 did not show any mutations in cervical and ovarian cancer samples or healthy individuals. An A1887G mutation and a G1891A mutation were detected in two and one samples of cervical cancer patients, respectively. The G1595T mutation was observed in 4 sequences obtained from patients with ovarian cancer. In addition, in one of the samples, the A1774T mutation resulted in an amino acid change. Details of mutations obtained from the DNA of cervical and ovarian cancer patients are listed in Table 2. In addition, there were no significant relationships between mutations and grades of the cancerous tissue (*P*>0.05), which might be due to the low number of samples and mutations.

**Table 2:** The frequency of EBNA1 C-terminal region variations in the cervical and ovarian cancer groups

		Cervical cancer		Ovarian cancer	
Nucleotide/Amino acid		**1887/629**	**1891/631**	**1595/532**	1774/591
Subtype	Sample				
B95	P-Ala	GAA/E	GGT/G	CGT/R	**AAT/N**
CC6	P-Ala	GAG/E	AGG/R	-	**-**
CC3	P-Ala	GAG/E	-	-	**-**
OC18	P-Ala	-	-	CTT/L	**TAT/Y**
OC22	P-Ala	-	-	CTT/L	**-**
OC24	P-Ala	-	-	CTT/L	**-**
OC27	P-Ala	-	-	CTT/L	

## Discussion

EBNA1 protein is a nuclear antigen essential for maintaining the episomal DNA of EBV in infected cells and is the only protein required for latent virus replication. Based on the amino acid sequence at position 487 of the EBNA1 protein, EBV is classified into three variants and two prototypes. In the present study, all sequences (from cervical and ovarian cancer patients + healthy individuals) had alanine amino acid at position 487 of the EBNA1 protein, and the EBNA1 prototype was P-Ala. In the same line, Gaal* et al.* showed that 9 out of the 12 NK/T cell lymphoma samples contained the P-Ala prototype (35). Also, Karbalaei* et al.* reported that no significant difference was observed between the EBNA1 subspecies and other variables (36). On the other hand, in the study by Zhang* et al.*, 51 out of 54 nasopharyngeal cancer samples in China were infected with the V-Val subtype (36).

Moreover, in southern China, the dominant subspecies was V-Val in both healthy individuals (71%) and those with gastric cancer (35%) (37). The V-Leu subspecies are more prevalent in Africa, and the P-Ala and P-Thr subspecies are predominant in Europe (31). Although more studies are needed to generalize this study to the Iranian population, our study and other studies showed that the target population in Iran, in terms of phylogenetic analysis, is most similar to the European population and strain B 95. Therefore, it can be said that these changes have more of a geographical basis. There is no clear relationship between different types of EBNA1 subtypes and the severity and extent of carcinogenesis. Due to the low number of samples in this study, we recommend more studies with a larger sample size to obtain the EBV subtype in Iran.

In our study, two mutations, including A1887G and G1891A, were found in the C-terminus region of EBNA1 in cervical cancer patients. Moreover, we found two mutations in ovarian cancer patients, including G1595T and A1774T. It is not clear whether these mutations alter or not the overall structure of the third EBNA1 protein. Furthermore, sequence analysis showed no mutations in the C-terminus region of EBNA1 in healthy individuals' specimens. 

Although in this study the number and type of mutations were higher in the cervical and ovarian cancer samples than in healthy ones, the difference was not statistically significant, indicating that mutations at the C-terminus region of EBNA1 might not be involved in the pathogenesis of EBV during cervical and ovarian cancer development. In addition, due to the low frequency of mutations in the C-terminal region of EBNA1 in this study, the genetic variation of EBV in cervical and ovarian cancer tissue samples seems to be low compared to the gastric and nasopharyngeal carcinomas associated with EBV (38-40). These findings suggested the possibility of the relative stability of the C-terminal region of EBNA1 sequences in cervical and ovarian cancer tissue, at least in comparison with gastric and nasopharyngeal carcinomas. In addition, due to the low number of mutations, there was no significant relationship between the frequency of mutations and the grades of cancerous tissue. Regarding the age of patients and healthy volunteer subjects, there was no statistically significant relationship between the frequency of mutation and the age of the subjects. 

To the best of our knowledge, there are few published data regarding the sequencing of the carboxylic region of the EBNA1 gene. In this regard, two studies by Chen* et al.* and Wrightham* et al.* reported mutations in the amino acid sequences of the carboxylic terminus of the EBNA1 gene (41, 42). Although we showed G631R and E629E variations in this region, they reported the G631D and E629D variations, respectively (41).

## Conclusion

In conclusion, the results of this study indicated that the EBNA1 prototype in cervical and ovarian cancers is P-Ala. Moreover, the frequency of mutation in the C-terminal region of ENBA1 indicated the relative stability of the C-terminus region of EBNA1 sequences that might not be involved in the pathogenesis of EBV in cancerous lesions of cervical and ovarian cancer patients. Due to the low number of subjects, more investigations are recommended to clarify these results.

## Conflict of Interest

None declared

## Ethical Approval

The study was approved by the Ethics Committee of the Shiraz University of Medical Sciences (IR.SUMS.REC.1399.948).

## Informed Consent

Informed consent was obtained before sample collection.

## Authors' contribution

Study concept: Sarvari J, Hosseini SY, and Safaei A; Sample collection: Safaei A, and Hosseini Tabatabaie F; Bench work: Hosseini Tabatabaie F, and Hashemi MA; Data analysis: Sarvari J, Hosseini SY, Hosseini Tabatabaie F, and Hashemi MA; Manuscript drafting: Hosseini SY, Hosseini Tabatabaie F, and Hashemi MA; Critical revision of the manuscript: Sarvari J, Hosseini SY, and Safaei A. All authors read and approved the final manuscript.

## Funding

This research was funded by Shiraz University of Medical Sciences.
